# Foreign Summary

**Published:** 1839-11-02

**Authors:** 


### FOREIGN SUMMARY.

### Velpeau's Clinical Lectures on Ophthalmia. No. VIII.

#### INFLAMMATORY AFFECTIONS OF THE CORNEA.

*Acute Keratitis, continued.*

The description I have just given of acute keratitis must have proved to you how extremely important it is that the treatment should be well understood. If, however, you consult what has been said on the subject by different authors, you will find that it yet remains a complete chaos. Indeed, nothing can more clearly show the utility—nay, the necessity—of studying the inflammatory affections of the eye, in the tissue which the inflammation attacks, than the confusion which reigns in the treatment of keratitis:

it is one of those diseases against which nearly every remedy has been in its turn tried, lauded, and then abandoned. With some practitioners every thing succeeds; with others, every thing fails. Some advocate general measures, to the exclusion of local treatment; others, again, uphold local applications as being the most beneficial. This diversity of opinion originates, no doubt, in the little attention which has been paid to the diagnosis of the different forms of keratitis, and to the opinions which nearly all ophthalmologists entertain respecting the specific nature of the disease. I do not flatter myself that I have solved this question, but I think I am now able to say, that in one case the disease may be cured, whereas in another a favourable termination is not to be expected—to say why a remedy succeeds, or why it does not succeed; otherwise the distinction I have established between the various forms of keratitis, by separately studying the inflammation of each part of the cornea, would be of no value; precision in the diagnosis of disease being only desirable as a means of throwing additional light on its treatment.

The treatment of acute keratitis, like that of most other diseases, is general and local.

*General treatment.*—When treating of the inflammatory affections of the palpebræ, I proved by a number of facts that little reliance, comparatively speaking, could be placed on general treatment, and that it ought only to be considered as an adjuvant to local measures. This cannot, however, be said of keratitis, against which general treatment stands pre-eminent, although it is still frequently necessary to resort to local applications. I shall now merely speak of the treatment of acute keratitis, setting aside every thing relating to the various phenomena by which it may be complicated.

The therapeutical agents which enter into the treatment of keratitis may be divided into two classes. The first comprises blood-letting and external derivatives; the other, purgatives and alteratives.

*Blood-letting.*—When the inflammation is intense, it is well to commence the treatment by general blood-letting. I need scarcely add, that the quantity of blood abstracted must be proportioned to the constitution of the patient, and to the state of his health at the time. Much discussion has arisen as to whence the blood should be taken—from the veins of the arm, or from the temporal artery. Judging *à priori*, arteriotomy would seem most likely to produce the desired effect. I have not myself resorted to this operation often enough to speak very decidedly on the subject: I must, however, say, that neither the cases in which I have practised it, nor the arguments brought forward in its favour, have yet convinced me of its superior efficacy. Some practitioners have also asserted, that when venesection is employed, the external jugular vein ought to be preferred to the veins of the arm. This appears rational; but when we consider how difficult it often is to obtain a sufficient quantity of blood from the jugular vein, and how important it is, at the same time, that the progress of the inflammation should be arrested, I think we cannot but prefer the usual plan of performing venesection in the bend of the arm.

Another important question is, the extent to which bleeding should be carried, and the manner in which it should be practised. I have made numerous experiments with a view to elucidate the point, and have come to the following conclusion. If the patient is young, robust, and of a plethoric habit of body, I bleed copiously, to the extent of eighteen or twenty ounces, and repeat the bleeding the next day; some times, also, the third day, as the inflammation, which at first rather subsides, might otherwise reappear. If, on the contrary, the patient is rather weak, I take a smaller quantity of blood at first, and repeat the bleeding at greater intervals. With patients of an ordinary constitution, bleeding night and morning, for several days, according to M. Bouillaud's plan, appears to be attended with beneficial results. General bleeding, in most cases, gives great relief. The following day you will nearly always find that the cephalalgia, the burning heat, the epiphora, and the photophobia, are much less intense; but they do not disappear entirely, and indeed often return. The loss of blood seems merely to act against the tendency of the system in general to inflammation; if no other measures are adopted, the disease either remains stationary, or assumes the chronic form.

Leeches are by no means so efficacious a remedy as general bleeding, yet in some instances they ought to be preferred; when, for instance, the patient is of a weak constitution, or of a lymphatic temperament, or when the circulating fluid has been impoverished, or its quantity diminished, by disease or by previous bleeding. When a patient is in this state, fifteen leeches applied behind the ear will have as beneficial an effect as copious general bleeding in a robust person. Leeches may be applied to the mastoid region, to the temples, to the inner canthus of the eye, or to the internal surface of the eyelids. I seldom, however, apply them to the inner canthus, as I am not aware that any great advantage is to be derived from this expedient, and in private practice they very frequently alarm the friends of the patient, by the swelling and infiltration they produce. Nor do I apply them to the internal surface of the eyelids, the relief they give to the patient being too slight to counterbalance the trouble they occasion.

The abstraction of blood by cupping may be classed along with leeches, and resorted to in similar circumstances. It is generally attended with beneficial effects when the inflammation tends to become chronic.

*Blisters, Setons, Moxas, &c.*—Blisters applied to the nape of the neck have been much employed in the treatment of keratitis. If, however, the effects they produce are minutely analyzed, we shall find that they exercise much less influence over the march of the disease than is generally supposed. Combined with other measures they may be useful, but alone they cannot cure; indeed, I am inclined to think that the application of blisters to the neck—the region which is generally chosen—does more harm than good. In some persons, especially children, they occasion a local reaction, which is followed by swelling of the lymphatic glands of the neck. When the keratitis is cured, the blister is allowed to heal, but the lymphatic system has been excited, and the swelling of the glands continues. In many instances I believe that children have been looked upon as scrofulous, although the presence of the characters which are supposed to denote this affection was to be attributed solely to such a cause. Little benefit, you see, is to be derived from blisters when employed in the usual manner; I wish, therefore, to direct your attention to a peculiar mode of applying them to which I have frequent recourse; I allude to the application of blisters over the eyelids themselves—a measure which is nearly always followed by the most beneficial results. This plan of treatment, which I ought, perhaps, to have described when speaking of local remedies, has been tried in purulent ophthalmia, but we have no satisfactory account of the essays which were made. At first it seems irrational, and even dangerous; when, however, we examine the subject more minutely, we find that it is neither in contradiction with practice nor theory. It was, indeed, from theoretical views that I was induced to make use of the remedy. Recollecting that I had seen obstinate cases of ophthalmia disappear under the influence of the irritation caused by an external affection, it occurred to me that a large blister over the eyelids might, by producing a kind of artificial erysipelas, be followed by the same results. I had already at that time studied the distribution of the vessels of the eye and of the eyelids, and was aware of the intimate connection which exists between them. Being anxious to ascertain the practical value of these ideas, I resolved to try the plan on the first obstinate case of ophthalmia that presented itself. This I had soon an opportunity of doing on an old soldier, who had just returned from Africa: both his eyes were violently inflamed, and I had a large blister applied over each. I was not, I must confess, quite easy with regard to the result of the step I had taken, and was consequently much pleased to hear him say the following morning that the pain had considerably abated. In two or three days I was able to open the eyelids, and then found that great amelioration had taken place. The eye continued to improve for about ten days: the affection then appearing to be stationary, I had again recourse to blisters, with the same beneficial results as before. This patient was not, however, cured; but we must not forget that he was labouring under one of the most obstinate forms of ophthalmia—keratitis, accompanied by granular conjunctivitis. Since then I have employed blisters over the eye more than a hundred times in every form of inflammation, principally with a view to ascertain when they ought to be used. I have found them an extremely useful remedy in conjunctivitis, in superficial and in deep-seated keratitis, and especially in ulcerated keratitis. The counter-irritation they produce appears to dissipate the congestion of the tissues, to arrest or prevent the effusion of coagulable lymph, and to modify the ulcerations, if any exist. They are not, on the contrary, very beneficial in blepharitis, purulent ophthalmia, in inflammation of the interior of the eye, nor even in keratitis when the inflammation has assumed the chronic form: in this case topical applications are the best remedies we can employ.

Some few years ago, when I wished to ascertain the comparative efficacy of this remedy in the different forms of ophthalmia, I employed it nearly indiscriminately; but now that I know in which form it is likely to prove the most beneficial, I have recourse to it much less frequently. Another reason which often deters me from applying blisters when I otherwise should, is the repugnance which patients, especially in private practice, manifest towards them. This repugnance is founded on the supposition that the application of a blister in this region will occasion great pain, and be followed with much disfigurement. The blisters themselves are, however, much less painful than is generally supposed, and the pain which was felt before nearly always abates in a very short time after they have been applied. If you question a patient with a view to ascertain the fact, you must remember that he is suffering two distinct kinds of pain—that caused by the blister, and that caused by the ophthalmia; you must, therefore, endeavour to make him distinguish between the two. The alarm which females generally show, from the supposition that the application of a blister over the eye will be followed by more or less disfigurement, is also without the least foundation, for the epidermis alone being destroyed, the skin soon loses the red tint which it has at first. I have seen persons over whose eyes four or five blisters had been applied successively, without the slightest trace of redness a few weeks after. Still, as these prejudices are difficult to overcome, I should not advise this plan of treatment unless you think it necessary.

When blisters are applied over the eyelids, some precautions are necessary; I will, therefore, tell you briefly what is to be done. The patient must close his eyelids gently, without contracting them, as they would otherwise be more or less folded, and the blister would not cover their entire surface. The blisters must be then carefully applied, in such a manner as to be in contact with every portion of the external surface of the palpebræ, and over it is placed some lint or cotton to fill up the cavity of the orbit, in order that compression may be equal. A bandage carried a few times round the head, and over the eye, will suffice to keep every thing in position. You will generally find the eyelids much swollen the next day, when the blister is taken away; it must be dressed as usual, without any attempt being made to see the eye. In the course of two or three days the swelling diminishes, the eyelids become hardened, and the eye may be freely examined. Although the pain generally diminishes in the course of a few hours, the amelioration produced by the blister is not very sensible for a day or two. The symptoms are then found to abate, and they continue to do so for about ten days, when it becomes after requisite to repeat the blister. It is necessary that you should be aware that the amelioration does not take place at first, as you might otherwise be led to despair of any effect being produced, and employ some other remedy, to which the subsequent improvement would be attributed.

In conclusion, blisters over the eyelids seldom effect a cure alone, but they nearly always diminish the violence of the disease, even when every other plan of treatment has failed to do so, and thus enable you effectually to overcome the malady. I have repeatedly found this the case in obstinate inflammatory affections of the cornea, accompanied by ulceration.

Many practitioners have of late endeavoured to substitute for the cantharides plaster some other substance capable of producing vesication, in order to avoid the action of the cantharides on the urinary organs. Various therapeutical agents have been tried, among which ammonia and tartarized antimony occupy the first rank; indeed, so much attention has been paid to this last remedy, that I cannot pass it over in silence. I have frequently employed it under the form generally used, that of an ointment. It gives rise to a crop of numerous small pustules, and the effect, as also that of ammonia, seems to me similar in every respect to that produced by blisters, on which, you know, I do not place much reliance, when applied in the usual manner.

Setons are often productive of benefit. The irritation they occasion in the subcutaneous fibro-cellular tissue appears to exert more or less influence over the inflammatory affections of the cornea, whereas blisters, which are applied to the skin, appear more especially to act on the affections of the conjunctiva. There is, indeed, some analogy between the fibro-cellular tissue, which is the seat of the irritation when a seton is used, and the tissue of the cornea, on the one hand, and the organization of the skin, and that of the conjunctiva, on the other. I do not know whether these theoretical ideas are correct, but the remark which preceded them is certainly justified by practice. Although setons sometimes give rise to erysipelas, and to abscesses, I have often recourse to them when the keratitis resists the action of other remedies.—*London Med. Gaz.*

---

*Observations on Yellow Fever.* By W. Ferguson, Esq., Surgeon in the Royal African Corps.—The susceptibility of yellow fever of being conveyed from one person to another, and from place to place, by means of the effluvium arising from the bodies of the sick, is a question which has of late years been occasionally agitated in your own and in other periodicals: the bulk of evidence and of authority appear to be ranged on the negative, although the affirmative side of the question has not been without its supporters.

The great magnitude and importance of the question, as well in its scientific as in its political bearing, calls loudly on every one who has had an opportunity of observing the progress of the disease, to contribute something to aid in dispelling the doubt and uncertainty which yet surround it, however obscure the individual, and however humble the contribution. Having had an opportunity of observing the progress of the disease at Sierra Leone on three different occasions, I therefore venture to submit my mite for consideration.

European residents have been many years settled at each of the following places along the coast of Western Africa, viz.:

| *Place.* | *By whom inhabited.* | *Latitude.* | | *Longitude.* | |
|---|---|---|---|---|---|
| River Senegal. | French. | 16° | N. | 16° | W. |
| Island of Goree. | French. | 14 | N. | 17 | |
| River Gambia. | English. | 13 | N. | 16 | |
| Cackeo. | Portuguese. | 12 | 10 | 16 | 23 |
| Island of Bissao, | Portuguese. | 11 | 51 | 15 | 37 |
| Rio Nunez. | English, French, Portuguese. | 10 | 36 | 14 | 42 |
| Rio Pongo. | English, French, Americans. | 10 | 7 | 13 | 58 |
| Sierra Leone. | English. | 8 | 30 | 13 | — |
| Accra. | English, Dutch, Danes. | 5 | 32 | — | 13 |
| Anamaboe. | English. | 5 | 10 | 1 | 7 |
| St. George Del Mina. | Dutch. | 5 | 5 | 1 | 22 |
| Island of Assension. | English. | 7 | 54 | 14 | 26 |

Of the above-named places epidemic yellow fever has appeared in the Island of Goree once (1837,) in the River Gambia once (1837,) at Sierra Leone four times (1823, 1829, and twice in 1837,) at the Island of Ascension twice, (1823, 1838.)

The disease is totally unknown, and has, I believe, never appeared at any of the other stations above named, and several of these have been colonized by Europeans one and two hundred years.

It is by no means a comfortable reflection to those whose lot is cast in such a land, that they incur a constant liability to such irruptions of disease as that which lately desolated Sierra Leone, against which neither prudence nor great care are of any avail as a safeguard; hence it is not to be wondered at, that, on former occasions of the epidemic irruption of yellow fever at Sierra Leone, there were many persons who sought out with great diligence every circumstance tending to show that the disease was not a sporadic, but an imported one. The evidence in proof of an object so desirable, entirely failed; and it is now, I believe, admitted by all classes at Sierra Leone, that on every occasion of its appearance there, it has been the undoubted product of the colony itself.

But while it appears thus clear that at Sierra Leone, the disease has never been an imported one: there are, on the other hand, facts sufficiently numerous and cogent to encourage a rather confident opinion, that, at every other part of this station where it has appeared, it has on every such occasion been of imported, not of sporadic origin.

Before going into details, it may be here stated generally, that wherever the disease has appeared on the West African station, at places other than Sierra Leone, such appearance has on every such occasion been preceded, within a very short time, by the arrival of a vessel having the disease on board, and the actual disembarkation of the sick at that place; and that the disease has never appeared at any part of the station, other than Sierra Leone, excepting after such arrival and disembarkation of persons labouring under it.

Though the observations which I am about to adduce on this much controverted matter are at variance with the opinions entertained by many persons whom I am bound to regard with much professional respect, yet I see nothing which ought to deter me from an honest, and I hope not a dictatorial or presumptuous, statement of my own experience : to this I will add the information which I have derived from sources worthy of credit, and the conclusions legitimately deduced from our united observations.

To begin with the Island of Ascension. It appears, as stated above, that epidemic yellow fever has prevailed there twice, viz. in 1823 and in 1838. With respect to its appearance there in 1823, it appears by the report of Dr. William Barry, then staff-surgeon, that yellow fever broke out at Sierra Leone in December, 1822, and prevailed, with more or less of severity, during the ensuing six months.

The Bann, sloop of war, arrived at Sierra Leone, (it does not appear when,) and departed on the 27th of March, with "three fever cases on board." Shortly after her departure, the fever cases accumulated so rapidly, that, instead of proceeding to the Island of St. Thomas, as was originally intended, she went to Ascension, and there debarked her sick.

The Bann had communication with the Driver, sloop of war, at Ascension; with what consequences to the garrison there, and to the crew of the Driver, is shown in the following extract of an official report of Assistant Surgeon, Sinclair, then serving in the Bann :—"Between her arrival (at Sierra Leone) and the departure of the Bann, for Ascension, on the 27th of March, several cases occurred, and the ship left Sierra Leone with three fever cases on board ; in a very few days the fever attacked so many, that instead of touching at St. Thomas, as was the original intention, for cutting wood, she was obliged to proceed to Ascension direct; she arrived there on the 25th April, but the fever had already committed such ravages on board, that scarcely a sufficient number of men were left to carry the sick on shore.

"The Driver arrived at Ascension on the 2d May, at that time very healthy, as well as the garrison on shore; but the Bann had already buried 32 men. All intercourse between the garrison and the sick tents of the Bann, was forbidden, and as much as possible between the men of the Bann and those of the Driver.

"In a few days an admiralty clerk, belonging to the Driver, sent on board the Bann to assist at a survey, and Captain Sawmarez and his servant, sent to join the Bann, were all seized with fever. About the same time the fever made its appearance among the garrison ashore, in the family of a soldier's wife, who had been washing for one of the Bann: it first seized a boy, and then the woman herself, and in a few days four men belonging to the garrison were attacked. Of the crew of the Bann, consisting of about 130, not so many as ten escaped fever, and 38 died; and of the Island of Ascension, the garrison consisting of 36 souls, five only escaped fever, and 17 died ; and of about eight from the Driver, who were exposed te the contagion, four were seized, and three died."

The disease did not again appear at the Island of Ascension until 1838. My information is not sufficiently precise as to the date of its breaking out; however, the events by which that irruption was preceeded, have been sufficiently ascertained

H. M. brigantine Forester, arrived at Sierra Leone from England, on or about the 5th December, 1837, and remained there four or five days, a time when epidemic yellow fever prevailed extensively in the harbour; the disease broke out among the Forester's crew a few days after she left Sierra Leone, on which she proceeded to the Island of Ascension. Lieutenant Rosenberg, (the commander,) and several of the crew, died before the vessel reached that place.

On her arrival there, the sick were disembarked at Comfort Core, a place variously reported to me as being from one to three miles from the barracks, where the garrison is quartered.

A rigid quarantine was established on the sick at Comfort Core.

The wearing apparel of the deceased commander, Lieutenant Rosenberg, was, I have been told, taken on shore, and there sold by public auction in the garrison,—the clothes, however, had been previously well and thoroughly washed on board. The disease broke out in the garrison about four weeks after the Forester's arrival, and proved fatal to the commandant, a medical officer, and many of the marines. I regret that dates and numbers are wanting in this statement; the correctness of the main fact may, however, be confidently relied on, viz.: that the disease on this, as on the former occasion, did not appear among the garrison at Ascension until after the arrival of a sickly ship, and the actual debarkation on the island of persons labouring under the disease.

These two are, I believe, the only occasions on which the disease has appeared at Ascension; they are, also, I believe, the only occasions on which persons labouring under the disease have been landed there.

With respect to its mode of introduction at the Gambia.

H. M. brig Curlew, having been appointed to cruise on the windward part of this station, was several times in the harbour of Freetown, Sierra Leone, in May, 1837, a time when epidemic yellow fever prevailed there; she left Sierra Leone to proceed to Gambia about the middle of May, and a few days thereafter, while on the passage, the disease broke out among the crew. She arrived at Bathurst, on the Gambia, on the 4th June, and Mr. Tebbs, the Colonial surgeon, who was at that time also in medical charge of the troops, had the sick all removed from the vessel, and taken, not to the hospital, but to his own house, the ground floor of which he had fitted up as an hospital for merchant seamen. Fifteen of the crew died.

Mr. Tebbs, who is reported to have been most diligent in his attendance on the sick, was laid up with the disease on the 17th, and died on the 20th June. An European boy, who had assisted Mr. Tebbs some years as a dispenser of medicines, and whose conduct is also spoken of in terms of high praise, followed his master to the grave in a few days, having been cut off by the same disease. Thirty-three days after this the disease appeared in the house next to that of Mr. Tebbs on one side, and immediately thereafter in the house next to that of Mr. Tebbs on the other side: it then followed an eccentric course over the town, and carried off more than one-half of the European population.

The Island of St. Mary, in the River Gambia, has been colonized by Europeans twenty-two years. I have conversed there with several intelligent Europeans who have resided either at Gambia, Senegal, or Goree, uninterruptedly, upwards of thirty years, and by them I am assured that they never either witnessed or heard of the disease at Gambia until the period of the Curlew's arrival there.

The total absence of any mention of the disease in the army medical returns or reports from that part of the station, serves to corrobate these statements.

It is clear, then, that at the Gambia, as at the Island of Ascension, epidemic yellow fever has never appeared but within a short time after the arrival and debarkation at the settlement of persons laboring under the disease, and that this was the only occasion on which persons laboring under the disease have been landed here.

Epidemic yellow fever having, as above stated, appeared at the British settlement in the River Gambia, and having for some time successively carried off every one whom it attacked, great consternation and alarm were in consequence excited, and several persons, who had it in their power to do so, departed from the place.

Mr. Heddle, a respectable merchant of Bathurst, was among those who migrated. He left Bathurst for the Island of Goree on the 9th of August, accompanied by Mr. Stubbs (an English gentleman) and Mons. Imbert, a resident of Goree, all in good health: the vessel arrived at Goree on the 12th.

Mons. Imbert was attacked with fever on the passage, and was landed in that state at Goree, on the 12th; he was taken to his mother's house, and by her he was attended in the most assiduous and affectionate manner until he died. The separation of mother and son was, however, of short duration; for, the same day on which Mons. Imbert died, his mother was attacked with fever, of a similar description, of which she died in four days.

Meantime, Mr. Stubbs, the other passenger, was attacked with fever on the 12th, (the day of arrival at Goree,) and died on the 16th.

Mr. Forster, a respectable merchant of Bathurst, left Gambia for Goree on the 17th August, accompanied by Mr. A. Hunter, the Colonial Secretary. Mr. Hunter was attacked with fever on the 19th, which terminated in "black vomit" and death, at Goree, on the 21st.

Between three and four weeks after the occurrences herein detailed, epidemic yellow fever broke out at Goree, and carried off a vast number of the population.

Goree has been colonized by Europeans upwards of 90 years, and it does not appear, either from written record or oral tradition, that the island was ever visited by yellow fever before.

It is hence as clear as any available evidence can make it, that, like the Island of Ascension and the British settlement on the River Gambia, the Island of Goree has only been visited by epidemic yellow fever on occasion of the arrival and debarkation there of persons laboring under the disease.

The negative evidence afforded by the progress of the disease on this station appears to lead to conclusions precisely similar to those deducible from the positive evidence.

To begin again with the Island of Ascension. It has been shown that epidemic yellow fever broke out at this place in 1823, and again in 1838, on each occasion shortly after the arrival and debarkation there of persons laboring under the disease. On each of those occasions (if the views herein developed are correct) the disease was conveyed to Ascension from Sierra Leone, but the disease also prevailed epidemically at Sierra Leone in 1829, on which occasion, although several vessels of the squadron carried it away from that place, it does not appear that in that year any person laboring under it was disembarked on the island. In that year the island wholly escaped its ravages.

The fortresses on the Gold Coast have been colonized, I believe, since the middle of the 17th century: it does not appear that epidemic yellow fever has ever been known there, neither does it appear that persons laboring under the disease have ever been landed there.

The several places at which Europeans reside betwixt Sierra Leone and the River Gambia (some of which, such as the Island of Bissao, have been colonized upwards of two hundred years,) have never, so far as I can learn, been visited by yellow fever.

I conversed on this subject, at the Island of Bissao, with several of the oldest and most intelligent Portuguse residents, and was assured that neither while the disease prevailed at Sierra Leone in 1823, 1829, 1837, or 1838, nor on any other occasion, has it ever appeared at that island.

It is, I think, difficult to account for the total exemption from the disease enjoyed by those places, and its appearance at Gambia and Goree, on any other supposition than this, that those intervening places have never been visited by persons laboring under it.

Ascension, on the one hand, and Gambia and Goree on the other, are the extremities of lines radiating from a centre at Sierra Leone; and were the epidemic appearance of the disease at those extremes to be accounted for by the influence of a generally pervading atmospherical cause, wherein lies the cause of exemption at the numerous intervening stations? and by what means did the disease at once jump from the centre to the circumference?

The connecting links in the chain, from the nucleus of the disease at Sierra Leone to its development at Ascension, Gambia, and Goree, appear to me to have been on every occasion sufficiently obvious, continuous, and unbroken, to render the other mode of accounting for its appearance at those places exceedingly probable, and in the present undecided state of positive knowledge on the subject, that which is least beset with difficulties.

The disease, as has been shown, having been conveyed from Sierra Leone to Gambia, and thence to Goree, occasioned the greatest alarm at the French settlement of Fort St. Louis, on the Senegal; the authorities there acting on the supposition that both at Gambia and Goree the disease had been imported, established a rigid system of exclusion on all vessels from either of those places so long as the epidemic should continue.

The settlement on the Senegal was not visited by the epidemic, neither has that settlement, so far as I can learn, ever been visited by persons labouring under the disease.

It may be considered necessary that some proof should be offered as to the perfect identity of the disease stated to have at times prevailed at the different places above mentioned, and to have been conveyed from one place to another.

With the means of information which I possess, it may be difficult to accomplish this, otherwise than by the evidence afforded by the occurrence of the symptom called "black vomit." This symptom is by no means invariably developed in every case—not even in such cases as terminate fatally—but wherever, on this station, yellow fever has prevailed in a district, the occurrence of that symptom has been sufficiently frequent to stamp a character on the epidemic not easily to be mistaken—and that symptom was of very frequent occurrence in each of the several epidemics stated above to have been identical with the parent yellow fever of Sierra Leone.

I must in candor state, that the views adduced in this paper have been dissented from by several of my professional friends of the sister service, now, or lately serving on this station, while others have agreed with me.

It has been stated to me that the last irruption of epidemic fever at Ascension, (1838,) was caused by an extraordinary accumulation of mud and filth in a pit, after an unusually heavy fall of rain. Without questioning the efficacy or the competency of such a cause, I may merely observe, that it is somewhat singular that the power of the mud-pit to generate epidemic yellow fever, should have remained dormant until after the actual importation into the island of that disease by the sailors of the Forester; but granting that the pit full of mud and filth at Ascension was the real cause of the late epidemic at that place, the origin of the epidemics at Gambia and Goree still remain to be accounted for, if that which I have assumed as the correct, or at all events the most probable mode of accounting for their origin, is considered mistaken.

It will be observed that at Ascension, at Goree, and at Gambia, a period of three or four weeks always elapsed between the landing of the sick and the epidemic outbreaking of the disease among the population,—a degree of uniformity worthy of remark, whether the conclusion at which I have arrived be mistaken or not.

*Sierra Leone*, 10th May, 1839.

*Lond. Med. Gaz.*

---

*Coagula in the Cavities of the Heart.*—The very great frequency with which coagula are found in the cavities of the heart renders their history a subject of great interest; and however difficult it may appear to be to reconcile the possibility of continued circulation with their presence, yet pathological research has put the question beyond doubt. We see proofs of this:—

1. In the firmness of some coagula, which, indeed, are firm in a degree that is not compatible with post-mortem formation.
2. In their extremely close adhesion to the surfaces of the heart.
3. In the evident organization they have undergone.
4. In their occasionally containing fluids and substances the indubitable result of vital action.

The disposition to become firm and united to surfaces with which they happen to be in contact, is strictly in accordance with the proneness evinced on other occasions by the living surfaces to form adhesions.

The disposition to organization is in conformity with the tendency observed in blood at rest in the living body to become vascular; and it is by no means uncommon to meet with coagula presenting such condition, wherever difficulty to the free transit of blood exits. If, in consequence of irritation on the lining membrane of an artery, for instance, coagulable lymph is thrown out, the blood, in passing over this uneven surface, will have a constant tendency to form depositions; we see this where the internal membrane has lost its smoothness from any cause; and it is exemplified very fully in the collections with aneurysmal enlargements. Within the cavities of the heart numerous seemingly unimportant deviations from the natural state may, by impeding the free passage of the blood, produce obstructions which ultimately give rise to coagula; sometimes we find an inherent abnormal tendency in the blood itself to form coagula. That coagula should contain substances not originally included within them may, at first, appear not to be in accordance with the general laws of physiology; but the fact being established, that they admit of organization, and transmutation into substances possessing all the means of nutrition and growth, there remains no difficulty in believing that new depositions may partake of new characters, and constitute formations different from those in which they originated.

The progress of formations within the heart has been accurately described by Lænnec, under the name of vegetations; at first they resemble an amorphous mass of fibrine; they afterwards become supplied with vessels; and, finally, assume a determinate form. It is not necessary, however, that I should occupy your attention with reference to these productions, further than to explain the evidence upon which we may conclude that they exist during life.

M. Bouillaud gives an example of a soft coagulum that almost filled the right auricle of the heart; it was traversed in almost every direction by an infinite number of minute vessels; and of another, in which the right auricle and right ventricle contained masses of albuminous and fibrinous matter, evidently organized, and connected with the parietes of their respective cavities by filaments, which it was necessary to tear before they could be detached.

These concretions may contain pus, and cartilaginous and osseous substances, under such circumstances that it is impossible to account for their presence otherwise than by admitting that they have been formed within the coagulum. *Dr. Chowne on Pseudo-Morbid Appearances.—Lancet.*

---

*On the apparent lengthening or shortening of the Limb in Injuries and Diseases of the Hip.*—M. Sedillot, one of the surgeons of the Val-de-Grace Hospital at Paris, has published a very valuable practical paper on the difficulties of diagnosis in some cases of injury about the hip-joint, which we now propose to condense for the benefit of our readers.

He remarks—"The deviations of the pelvis, so common in diseases of the hip-joint, have been studied with great care of late years; and it is now admitted by all surgeons, that the *apparent* elongation of the limb on the affected side is generally owing to this cause (pelvic deviation) and not, as was formerly supposed, to a partial dislodgment of the head of the bone from its socket. It has been also ascertained that the deviation is not always from above downwards, but is occasionally from below upwards; in which latter case the limb, instead of seeming to be elongated, appears to be shortened; although, in truth, its real length is not altered.

The attention of surgeons having been thus directed to these points, a great improvement in the history of hip-joint disease has been introduced of recent years; but the pelvic deviations, caused by a sudden blow or contusion upon the haunch, have been rather neglected; although they deserve particular consideration, seeing that such accidents are exceedingly apt to be mistaken either for fractureof the pelvis or of the cervix femoris, or for some dislocation of the hip-joint—mistakes which might prove most serious both to patient and surgeon.

The following case may be adduced in proof.

A young robust man fell upon his right haunch and hip while coming down a flight of stairs; his right foot being caught under the balustrade, prevented him from slipping down the stairs. He heard a sharp crack at the time, and he thought that he must have broken his limb. He rose, however, without assistance, but he could not rest the foot of the injured side upon the ground. A surgeon, who was immediately called to his assistance, finding that the limb was shortened and could not be moved but with difficulty, considered the case as one of dislocation of the hip upwards and outwards; extension was therefore employed for some time, but without any benefit.

Next day a consultation was held; the patient lay on his back; his right thigh was somewhat bent upon the pelvis, and drawn out or abducted from the other, so that the two knees were about a foot apart; the right leg was bent upon the right thigh and drawn inwards, the heel being on a level with the middle of the left leg. While in this position the patient was free from pain; but he could not change it without great suffering.

Some of the medical men considered the accident to be a dislocation, while others regarded it as a fracture of the neck of the thigh-bone. It was therefore necessary to study the various symptoms with peculiar care.

When the two limbs were extended and brought together, the right one was found to be about an inch and a half shorter than the left one; no traction could bring the heels on a level with each other. When the knees were bent upon the thighs and brought together, the same degree of shortening of the right thigh was observed, in whatever position the pelvis was attempted to be placed. The great trochanter on the right side was more elevated than that on the left; the superior and anterior spinous process of the right *os ilii* also was an inch and a half, or so, higher than the corresponding process on the other side. Wherever therefore the measurement was taken, the right limb always seemed to be shorter by upwards of an inch than its fellow. The distance of the right trochanter, however, from the crest of the *os ilii* was observed to be uniformly the same, whatever was the position in which the limb was placed.

With respect to the direction of the foot on the injured side, it was, as already mentioned, rotated somewhat outwards; but the patient still retained the power of rolling it round and even inwards. He could also, while lying in bed, extend and raise the entire limb. The flexion of the thigh on the pelvis could not be carried beyond a right angle; adduction was difficult, but abduction was free and without uneasiness.* In the upright position, the patient rested rather on the heel than on the toes of the affected limb. The right shoulder was observed to be decidedly lower than its fellow, and the left side of the body was *legerement arqué*.

The patient experienced considerable pain when pressure was made over the hip-joint, and when the limb was rolled inwards and adducted; but very little when abducted. When he lay flat on his belly, the line of separation between the buttocks was sensibly inclined from right to left, and from below upwards.

Such were the most conspicuous features of the present case; and the question now came for consideration, what was the real nature of the injury present?

That the shortening of the limb was not owing to fracture of any part of the thigh-bone, M. Sedillot inferred from the circumstance that, although the foot on the injured side was drawn up about an inch and a half or so, and thus the limb *seemed* to be shorter than its fellow, the distance between the knee-joint and any point of the pelvis was exactly the same on both sides; this could not possibly be so, if one thigh were in reality shorter than the other. The distance too between the trochanters and the ant. sup. spinous processes of the ilium was steadily the same.

The free mobility of the limb also, although when left to itself it was turned outwards, and the power which the patient had of moving it in several directions, were arguments against the idea of fracture of the cervix femoris being present. Still it should be well remembered by the surgical reader that, in some cases of such fracture, the patient has at first, and even for several days after the accident, retained the power of moving his limb, nay, has even walked for some distance, and yet the distinctive characters of the injury—viz., shortening of the limb, rolling of the foot outwards, crepitation when the limb is extended and turned round, pain, &c.—have not made their appearance for a day or two. The explanation of such an occurrence is probably to be sought for in the circumstance that the fractured ends of the bone remained in contact at first, and that they were not separated until either the investing periosteum was torn, or the surrounding muscles had began to contract more powerfully than before.

* During these movements, a rubbing noise, *un bruit de frottement*, was audible; it was considered by some to be the crepitation of two broken surfaces, but M. Sedillot was of opinion that it was owing rather to the friction of fibrous surfaces one upon the other, or to slight articular shocks.

The age, too, of the patient, in the present case, naturally suggested a suspicion that the neck of the bone was *not* fractured. Of 225 cases of this accident, alluded to by Sir Astley Cooper, in two only was the age of the patient under fifty years.

M. Sedillot therefore felt satisfied that the case was not one of fracture. That it was not one of dislocation, he inferred from the circumstance of the distance between the great trochanter—although it was very projecting—and the crest of the ileum being the same on both sides, from the comparative facility of movement, more especially that of abduction, in the limb, from the trochanter not describing a larger arc than usual when the limb was rotated, and from the turning of the foot outwards.

Dwelling upon these and other considerations, he came to the conclusion that the case was merely one of severe contusion inducing a temporary lateral deviation of the pelvis. We have already stated that, whenever the hip is the seat of pain, the pelvis on the affected side very generally becomes elevated and inclined over somewhat to the opposite side, for the purpose, no doubt, of relieving the weight of the limb, and of the pressure of the foot on the ground. The shortening of the limb in such a case is only apparent; and this fact may always be readily ascertained by means of measurement with a piece of tape, as already alluded to.

It is not improbable that, along with the contusion of the pelvis, there was also a sprain of the hip-joint; perhaps some of the ligamentous and muscular fibres around it partially torn, or even a portion of its articular cartilage broken off. When such accidents are present, the symptoms of the case will necessarily be more obscure, and will simulate more exactly those of dislocation of, or fracture near to, the joint.

We have nothing to say as to the treatment of such injuries, as this must, as a matter of course, consist in rest, the application of leeches, and so forth. Should the deviation of the limb continue, after the immediate symptoms of the accident are removed, it may be necessary to resort to the use of mechanical means.—*Med. Chirurg. Rev.*, *from L'Experience.*

---

*Dr. Cadogan.*—This physician, who was at one time in indifferent circumstances, married a rich old lady, over whose wealth he had an entire control. Like most mercantile marriages, it was not of the happiest kind. The lady had a suspicion on her mind, that the doctor would one day poison her with his physic in order to get her out of his way; and feeling ill on one occasion she exclaimed that she was poisoned. "Poisoned!" said the doctor, to a number of his wife's friends who were present, "how can that possibly be? Whom do you accuse of the crime?" "You," replied the indignant wife. "Gentlemen," said the doctor, with considerable *non chalance*, "it is perfectly false. You are quite welcome to open her at once, and you will then discover the calumny."—*Physic and Physicians.*

